# Origin of the Word Tobacco

**Published:** 1811-06

**Authors:** 


### *To the Editors of the Medical and Physical Journal.*

Gentlemen,

I WAS much amused, as well as instructed, with the excellent paper of Medicus on Tobacco, inserted in a former number of your Journal ; such communications are creditable to your publication, and prove highly interesting to practitioners, who, like myself, cannot command an extensive library. I did not observe, however, that your correspondent offered any remarks on the origin of the word Tobacco, which is involved in some obscurity. Some authors have supposed that the plant derived its name from the island Tobago. Labat asserts that it was named from Tabasco, a town in New Spain. The most probable origin of the name is is from the pipe which was used in smoking being called Tobaco. The aborigines of Cuba were accustomed to celebrate a victory by a general dance, at the end of which they seldom failed to make themselves inebriated with the fumes of tobacco. Charlevoix, in his history of St. Domingo, relates that they do not begin to smoke, till ready to fall from fatigue in dancing. They then lay some leaves of the tobacco plant, not quite dry, upon some hot embers; they then take a forked pipe in the shape of Y; the extremity of this instrument is placed amidst the smoke which ascends, and the two branches are put into the nostrils of the smoker, who very soon becomes intoxicated; and this instrument is called Tobaco. The Spaniards say *Hazer un Tabaco*, to signify the diversion which they take in dancing and smoking in a circle like the Americans; and it is very probable that the term *Tabagie* as much used by some of the old French writers, to express the feasts of savages, was thence derived.

I am, Gentlemen,

Very respectfully, your Friend,

S. L.

*London*, *April* 19, 1811.

